# Incidental Scleral Icterus in an Adolescent Male With Nausea: Clinical Diagnosis of Gilbert Syndrome in the Pediatric Outpatient Setting

**DOI:** 10.7759/cureus.43298

**Published:** 2023-08-10

**Authors:** Alex Gilman, Vishaka R Hatcher, Donald Hefelfinger

**Affiliations:** 1 Pediatrics, Wright State University Boonshoft School of Medicine, Dayton, USA; 2 Pediatrics, Wright Patterson Medical Center, Dayton, USA; 3 Medicine, Wright State University Boonshoft School of Medicine, Dayton, USA

**Keywords:** hepatobiliary, jaundice, dyspepsia, hyperbilirubinemia, gilbert’s syndrome

## Abstract

Gilbert syndrome is a condition of non-hemolytic hyperbilirubinemia without further sequelae or primary laboratory abnormalities. Gilbert syndrome represents the most common hereditary disorder of bilirubin metabolism and is frequently identified as the etiology of familial jaundice in clinical medicine. This disorder typically manifests as mild unconjugated hyperbilirubinemia of benign nature. The diagnosis of Gilbert syndrome entails clinical assessment corroborated by the laboratory findings above in the absence of hemolysis or other organic liver diseases. We report a case of a 17-year-old boy who presented to a pediatric clinic with dyspepsia for the management of digestive symptoms, with clinical findings of mild scleral icterus and laboratory findings of isolated indirect hyperbilirubinemia. This case is unique in its subtlety of presentation. It highlights to trainees and experienced physicians the importance of the physical examination and targeted laboratory workup to arrive at the diagnosis of Gilbert syndrome.

## Introduction

Jaundice is a significant clinical symptom that is typically implicated with diagnoses of hyperbilirubinemia due to liver disease or hemolytic etiology [1-6]. Among the myriad of causes of hyperbilirubinemia, Gilbert syndrome is a well-established condition with the distinguishing feature of isolated elevated indirect (unconjugated) bilirubin levels with otherwise unremarkable hepatobiliary laboratory tests. Physiologic stressors that can precipitate the symptoms of underlying Gilbert syndrome include viral illnesses, fasting, malnutrition, dehydration, menstruation, and sleep deprivation [3-5,7]. Physical examination findings of jaundice or scleral icterus are often the only indicators of elevated levels of unconjugated bilirubin. With a high index of suspicion and presence of physical findings, a negative history can be used to rule out stressors which can help in further management. Most cases of Gilbert syndrome do not require significant medical intervention aside from management of the triggering stressor [7-9]. We present a case of a 17-year-old boy with ongoing symptoms previously attributed to dyspepsia, with pertinent clinical findings of mild scleral icterus and laboratory evaluation revealing an isolated unconjugated hyperbilirubinemia.

## Case presentation

A 17-year-old boy presented to our outpatient pediatric clinic with approximately six weeks of ongoing nausea, stool changes, and decreased appetite. Upon initial evaluation at symptom onset, he was diagnosed with dyspepsia and prescribed a proton pump inhibitor (PPI). The patient discontinued using PPI shortly after initiation, with a reported plateau in symptom relief. He was taken to the emergency department after approximately six weeks of progressively worsening nausea, diarrhea, and perceived weight loss. A review of systems was positive for intermittent daily nausea and diarrhea with some noticeable mucus occurring up to five times per day. The patient denied vomiting, fevers, chills, headaches, hematochezia, and melena. He denied changes in diet or travel before the onset of his symptoms. He endorsed no postprandial abdominal pain or other temporal trends in symptoms. The patient reported normal urinary frequency with no dysuria, hematuria, urinary urgency, or flank pain.

On physical examination, the patient was afebrile with normal vital signs. He was well-appearing and well-groomed with a slim build and BMI at the 15th percentile for his age and sex. His mood was euthymic without a flat affect. His abdomen was soft, non-distended, and non-tender, with active bowel sounds. With the exception of mild bilateral scleral icterus, all other physical examination findings were normal. Laboratory tests in the emergency department revealed elevation of indirect bilirubin without elevation in transaminases, lipase, or alkaline phosphatase (Table 1).

**Table 1:** Significant laboratory results pertinent to the patient workup during outpatient evaluation. Laboratory results were significant for elevated indirect bilirubin. The rest of the laboratory results were unremarkable. The total bilirubin of 2.5 mg/dL and indirect of 2.7 mg/dL were not anomalous. The laboratory discrepancy was explained by new machinery detecting indirect and direct bilirubin with greater accuracy than the standard metabolic panel. With this laboratory discrepancy, the indirect bilirubin was confirmed to be 2.7 mg/dL, and the direct bilirubin was confirmed to be 0. *There were normal WBC counts for different types of WBCs.

Lab category	Result	Units	Ref. range
Bilirubin	2.50 H	mg/dL	0.2-1.3
Bilirubin indirect	2.700 H	mg/dL	0.0-1.1
Bilirubin direct	0.00	mg/dL	0-0.3
Alanine aminotransferase	16	units/L	7-56
Aspartate transaminase	20	units/L	10-40
Hemoglobin	17	g/dL	14-18
Hematocrit	48.5	%	41-50
Mean corpuscular volume (MCV)	87.6	fL	80-100
Mean corpuscular hemoglobin (MCH)	30.8	pg/cell	27-31
Mean corpuscular hemoglobin concentration (MCHC)	25.1	g/dL	32-36
Red cell distribution width (RDW)	12.8	%	11-15
Platelets	235	-	150-450
White blood cells (WBC)	6000 w/normal difference*	WBCs/mL	4500-11,000
Prothrombin time	13.8	Seconds	10-13
International normalized ratio (INR)	1.17	-	1.1 or below
Partial thromboplastin time (PTT)	33.7	Seconds	60-70

Complete blood count was within normal limits, and coagulation studies were also noncontributory, with a mild elevation in prothrombin time (PT) that may be attributed to processing error given the otherwise unremarkable hematologic and hepatic laboratory results.

Gilbert syndrome was suspected and proposed as part of the differential diagnosis, along with gastroesophageal reflux disease, peptic ulcer disease, malabsorptive gastrointestinal disorders, irritable bowel syndrome, inflammatory bowel disease, and malignancy. Liver disease and hemolytic processes were also included in the differential due to the patient’s mild scleral icterus. With overall unremarkable hematologic and hepatic laboratory studies, the lack of pallor, petechiae, or bleeding on the examination was reassuring against an underlying hemolytic process or liver disease. The new onset of dyspepsia, associated decrease in caloric intake, weight loss, and stress increase indicate a presentation of Gilbert syndrome with characteristic laboratory findings and scleral icterus. Given the subtlety of his physical examination with isolated mild scleral icterus, similar manifestations of Gilbert syndrome at a younger age may have gone unrecognized and undiagnosed.

The patient’s nausea was effectively treated with ondansetron in the emergency department. Treatment of dyspepsia was resumed upon follow-up in the pediatric clinic when the family received education on the differential diagnosis of Gilbert syndrome. Plan was also made to repeat laboratory studies when symptoms resolve to assess not only the bilirubin level but also the PT, which was subtly elevated on initial evaluation. The patient was referred to gastroenterology for further evaluation of potential sequelae of uncontrolled dyspepsia and potential treatment if indicated.

## Discussion

An estimated 3-7% of Americans have Gilbert syndrome. The disease is more prevalent in males than females while exerting its effects across all ages and ethnicities. It is associated with an identified mutation in the UDP glucuronosyltransferase family 1 member A1 gene (UGT1A1), which codes for the enzyme UDP glucuronosyltransferase (UDPGT). UDPGT is responsible for the conjugation of bilirubin and allows normal clearance of bilirubin from the body. Patients with Gilbert syndrome exhibit this inherited mutation in the UGT1A1 gene, producing approximately 30% of the UDPGT needed by the body [8,9]. This underproduction of UDPGT results in reduced bilirubin conjugation and excretion. The excess bilirubin gets distributed into tissues via hematogenous spread. One of the first and most sensitive areas of bilirubin distribution is the sclerae, making scleral icterus the prevailing physical examination finding in patients [1-5].

Approximately 33% of patients with Gilbert syndrome remain asymptomatic, with diagnosis typically occurring via incidental laboratory findings of elevated indirect bilirubin [8,9]. Among those that are symptomatic, the most common sign is jaundice. Jaundice typically becomes detectable when serum bilirubin rises above 2-3 mg/dL, emphasizing the importance of identifying the subtle scleral icterus on our patient’s examination. Those who present with Gilbert syndrome can also experience dark-colored urine, acholic stools, generalized abdominal pain, nausea, diarrhea, anorexia, fatigue, dizziness, and fever [6-9]. Identified triggers of symptomatic jaundice in these patients include dehydration, fasting, malnutrition, stress, physical overexertion, and infection. Diagnosis is most commonly made in adolescence and early adulthood and is largely clinical. Confirmatory testing via enzymatic assessment of UDPGT content or genetic identification of UGT1A1 mutation is generally not indicated unless other hepatobiliary or hemolytic pathologies have not been ruled out [8,9]. This patient presented with signs of dyspepsia and gastritis but had a normal abdominal examination in the emergency department and in the pediatric clinic, therefore imaging studies were not obtained. If a patient were to present with scleral icterus, nausea, and tenderness or distention on abdominal examination, radiologic evaluation would be warranted prior to making a diagnosis.

Triggers of Gilbert syndrome can result in the recurrence of symptoms, therefore patient education for prompt identification supports timely management of the underlying triggers [7-9]. Generally, patients with Gilbert syndrome are not subjected to clinical interventions for jaundice itself. Counseling the patient on lifestyle management and limiting stressors that could trigger hyperbilirubinemia is beneficial.

## Conclusions

This study aimed to help clinicians better understand the presentation and diagnosis of Gilbert syndrome. While the differential diagnosis of our patient’s presenting symptoms started off broad, examination findings and laboratory testing narrowed the scope for the diagnosis of Gilbert syndrome. The pearl for clinicians from this case is that while dyspepsia, diarrhea, anorexia, and fatigue can raise concern for infectious and insidious etiology, targeted laboratory evaluation and thorough physical examination can provide diagnostic clarity for Gilbert syndrome without subjecting the patient to a battery of invasive testing and imaging studies. If a thorough history and physical examination raise concern for other acute or chronic intra-abdominal processes, further evaluation including imaging must be completed prior to making a diagnosis of Gilbert syndrome in order to exclude other possible etiologies. Being aware of the early signs and symptoms may help to diagnose patients with Gilbert syndrome earlier and help prevent recurrence with the management of known stressors.
